# Comparison of the Vessel Loop Technique Versus Traditional Skin Closure for the Prevention of Surgical Site Infection Following Laparotomy in General Surgery

**DOI:** 10.7759/cureus.112986

**Published:** 2026-07-20

**Authors:** Rosa Miranda Thais, Rafael Gaszynski

**Affiliations:** 1 General Surgery, Hornsby Ku-ring-gai Hospital, Sydney, AUS

**Keywords:** midline laparotomy, surgical site infection (ssi), urgent laparotomy, vessel loop, wound infections

## Abstract

Background

Surgical site infection (SSI) remains a common and costly complication following laparotomy. Passive subcutaneous drainage using vessel loops has been proposed as a simple method to reduce incisional SSI, but evidence in laparotomy patients is limited. This study aimed to compare the incidence of incisional SSI between vessel loop-assisted skin closure and conventional skin closure following laparotomy in general surgery patients.

Methods

A retrospective cohort study was conducted on adult patients undergoing laparotomy for general surgical indications between January 2020 and January 2025. Patients were grouped according to skin closure technique: vessel loop-assisted closure (Vessiloop) or conventional closure without vessel loops. The primary outcome was to compare the incidence of SSI in laparotomy patients who had vessel loop insertion compared to conventional laparotomy closure. The secondary outcomes were to evaluate the association between patient comorbidities, surgical type, and the risk of SSI. Statistical analyses were performed using IBM SPSS Statistics for Windows, Version 27.0 (Released 2019; IBM Corp., Armonk, NY, USA).

Results

A total of 70 patients were included: 35 in the Vessiloop group and 35 in the conventional closure group. Patients in the conventional closure group were older, had a higher ASA score, and had an increased rate of gastrointestinal disease. Overall, six patients (8.6%) developed SSI. No SSIs occurred in the Vessiloop group, whereas six patients (17.1%) in the conventional closure group developed SSIs (p = 0.025). Univariate analysis demonstrated associations between SSI and smoking; the small number of smokers limits the reliability of this observation. Return to theatre, readmission, and 30-day mortality were similar between the two groups.​​​​​​​

Conclusion

Vessel loop-assisted skin closure following laparotomy was associated with a lower incidence of incisional SSI in this retrospective cohort. However, baseline differences between groups may have contributed to the observed SSI rates. Larger prospective studies are warranted to validate these findings and define their role in SSI prevention.

## Introduction

Surgical site infection (SSI) following laparotomy is a common postoperative complication, typically occurring within 30 days of surgery. Abdominal procedures are associated with higher SSI rates due to intestinal bacterial load and the risk of intraoperative contamination. Previous studies have demonstrated that more than 80% of SSIs occur after hospital discharge, highlighting the importance of strategies that reduce postoperative wound complications beyond the inpatient setting [1,2].

Strategies to reduce SSI risk include optimisation of comorbidities prior to elective surgery, preference for minimally invasive approaches where feasible, adherence to perioperative antibiotic protocols, and implementation of postoperative surveillance programs [3,4]. SSIs are classified as superficial (skin and subcutaneous tissue), deep (involving fascia or muscle), and organ/space. The last is the most severe category and involves any anatomical site accessed during the procedure [5].

SSI imposes a substantial burden on the Australian healthcare system. Recent estimates suggest a direct annual cost exceeding AUD $320 million in public hospitals alone [6,7]. Moreover, SSIs contribute significantly to antimicrobial consumption, increasing the risk of antimicrobial resistance and associated morbidity [8]. Despite the widespread use of vessel loops in other surgical contexts, their role in preventing incisional SSI following laparotomy has not been systematically evaluated.

If vessel loop-assisted skin closure is shown to reduce the incidence of incisional SSI following laparotomy, it could represent a simple, cost-effective strategy to improve postoperative outcomes in general surgery. This cohort study has been reported in line with the STROCSS guidelines [9]. The primary objective of this study was to compare the incidence of SSI in laparotomy patients who underwent vessel loop insertion versus those who received conventional laparotomy closure. The secondary objectives were to evaluate the association between patient comorbidities, type of surgery, and the risk of SSI.

## Materials and methods

Study design and ethics

This retrospective single-centre cohort study was conducted at a metropolitan teaching hospital in Sydney, Australia (November 2025-February 2026) and approved by the Northern Sydney Local Health District Ethics Committee (2025/ETH02091) prior to commencement. De-identified patient data were extracted from electronic medical records; the ethics committee waived the requirement for individual informed consent. The study involved minimal risk to participants and no direct patient contact. All data were handled in accordance with institutional policies and relevant ethical guidelines to ensure patient confidentiality and privacy.

Patient selection

Inclusion Criteria

Adult patients (≥18 years) who underwent emergency laparotomy for closed-loop bowel obstruction, perforated diverticulitis with free gas, and elective incisional hernia repair were included.

Exclusion Criteria

**Figure 1:**
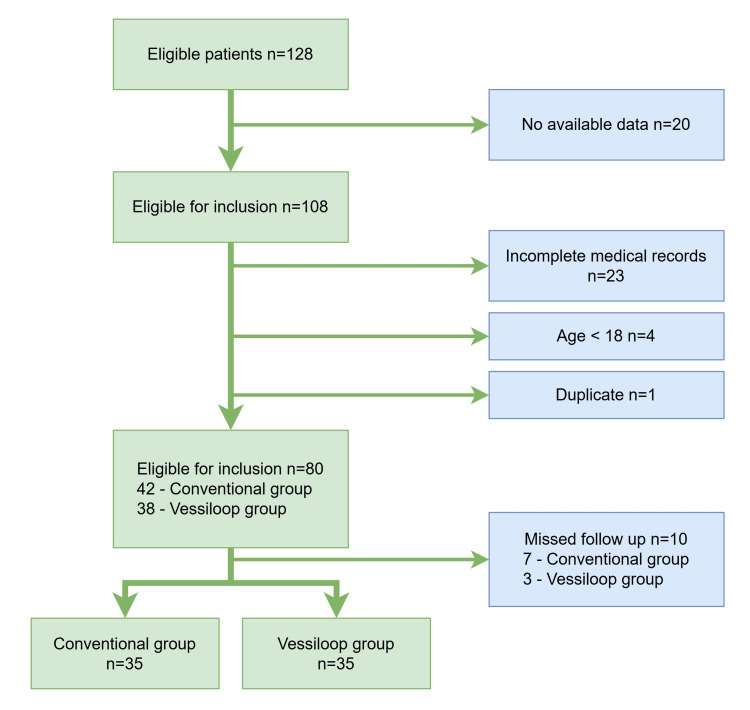
Participant STROBE flow diagram in laparotomy patients

Patients under 18 years of age with incomplete or missing clinical data or duplicate data were excluded. Please see the STROBE flow diagram in Figure 1 for further details.

Operative technique

The decision to use a subcutaneous vessel loop or a conventional closure was made at the discretion of the surgeon, based on clinical judgment and preference rather than predefined allocation criteria. We used two vessel loops, one on the proximal side of the wound and one on the distal side. We used only one vessel loop in patients undergoing mini-laparotomy. All patients received prophylactic antibiotics within 30 minutes prior to skin incision. Following standard skin preparation and draping, laparotomy was performed using a scalpel for skin incision and monopolar diathermy for subcutaneous tissue and linea alba division. A sterile wound retractor (Alexis wound retractor) was applied routinely. Fascial closure was performed using 0-0 monofilament absorbable suture (Maxon).

Vessiloop group

**Figure 2:**
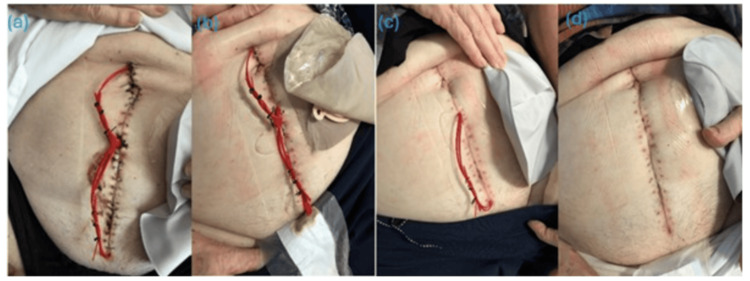
Postoperative images (a) Postoperative week 1: two Vessiloops were inserted into the proximal and distal portions of the wound and secured with 1/0 nylon sutures. (b) Serous yellow wound drainage was noted on the dressing. (c) Postoperative week 2: the proximal Vessiloop and skin staples were removed, while the distal Vessiloop remained in situ to facilitate ongoing drainage. (d) Following removal of the distal Vessiloop, the wound was clean, dry, and free of clinical signs of infection.

Following local anaesthetic infiltration, skin edges were approximated using staples or Monocryl sutures. Silicone vessel loops were placed between staples or sutures to allow passive subcutaneous drainage (Figure 2). Patients were reviewed in the outpatient clinic two weeks postoperatively for wound assessment and removal of staples and vessel loops.

Conventional closure group

Following local anaesthetic infiltration, the skin edges were approximated with staples or Monocryl sutures, without the use of vessel loops. Patients were reviewed in the outpatient clinic two weeks postoperatively for wound assessment and staple removal.

Outcome measures

The primary outcome was the incidence of incisional SSI within 30 days of surgery, classified as superficial, deep, and organ/space according to the Centers for Disease Control and Prevention (CDC) criteria. During hospital admission, the treating surgical team examined the wounds daily. Post-discharge surveillance was performed at a routine review two weeks after discharge in the outpatient clinic. SSI was verified through clinical documentation, biochemistry, and microbiology results. Patients who developed SSI were seen in the outpatient clinic earlier than their scheduled appointment. Some staples were removed from areas with signs of infection, and a swab was taken if purulent fluid was found; the staples in healthy areas of the wound were removed in week 2 post-surgery. This group of patients was referred to community nurses for regular wound assessment and was seen weekly in the outpatient clinic.

Statistical analysis

Continuous variable between-group comparisons were performed using independent-samples t-tests. Categorical variables were summarised as frequencies and percentages and analysed using chi-square or Fisher’s exact tests where indicated. The Clopper-Pearson interval was used to calculate the confidence interval. Statistical significance was defined as a two-sided p-value < 0.05. Given the retrospective design and non-random allocation of closure technique, results were interpreted as associative rather than causal. Multivariable adjustment was limited by sample size.

## Results

A total of 70 patients were included in the study. Thirty-five adult patients were treated with the Vessiloop technique (VT), and 35 with conventional laparotomy/no Vessiloop (NV). Patients in the conventional closure group were older, had a higher ASA score, and had an increased rate of gastrointestinal disease compared to the Vessiloop group. The distribution of sex, BMI, diabetes, obesity, cardiovascular disease, and other baseline characteristics was compared between groups. Baseline characteristics are summarised in Table 1.

**Table 1:** Baseline characteristics "+" denotes Fisher’s exact test, and "*" denotes the t-test. Categorical variables were compared using Pearson's chi-square test when the assumptions for the test were met. Fisher's exact test was used when expected cell frequencies were less than 5. Continuous variables were compared using the t-test. Statistical significance was defined as p < 0.05. GI, gastrointestinal; ASA, American Society of Anesthesiologists; DVT, deep venous thrombosis

Variables	Total (n = 70)	Vessiloop (n = 35)	No Vessiloop (n = 35)	p-value
Age (mean ± SD)	-	69.0 ± 16.3	77.8 ± 14.9	0.013*
Sex, n(%)				
Female	29 (41.4)	12 (34.3)	17 (48.6)	0.225
Male	41 (58.5)	23 (65.7)	18 (51.4)	
ASA (mean ± SD)	-	2.7 ± 0.7	3.2 ± 0.7	0.021*
Comorbidities, n(%)				
Diabetes	11 (15.7)	5 (14.3)	6 (17.1)	1.000^+^
Cardiovascular disease	20 (28.5)	7 (20.0)	13 (37.1)	0.112
Hypertension	30 (42.8)	12 (34.3)	18 (51.4)	0.147
Dyslipidaemia	15 (21.4)	6 (17.1)	9 (25.7)	0.382
Chronic kidney disease	5 (7.1)	2 (5.7)	3 (8.6)	1.000^+^
Liver disease	2 (2.8)	0 (0.0)	2 (5.7)	0.493^+^
Chronic lung disease	8 (11.4)	1 (2.8)	7 (20.0)	0.055^+^
Peripheral vascular disease	1 (1.4)	0 (0.0)	1 (2.8)	1.000^+^
Neurological disease	7 (10)	4 (11.4)	3 (8.6)	1.000^+^
Cancer	13 (18.5)	6 (17.1)	7 (20.0)	1.000^+^
Obesity	2 (2.8)	0 (0.0)	2 (5.7)	0.493^+^
GI disease	22 (31.4)	7 (20.0)	15 (42.8)	0.039
Previous abdominal surgery	19 (27.1)	6 (17.1)	13 (37.1)	0.060
DVT history	1 (1.4)	0 (0.0)	1 (2.8)	1.000^+^
Steroid use	1 (1.4)	1 (2.8)	0 (0.0)	1.000^+^
Immunosuppression	13 (18.5)	4 (11.4)	9 (25.7)	0.218^+^
Smoker	4 (5.7)	1 (2.8)	3 (8.6)	0.614^+^

The most common indication for laparotomy was small bowel surgery, followed by colorectal surgery. Rates of other postoperative complications, including pneumonia, sepsis, venous thromboembolism, return to theatre, readmission, and mortality, were low and did not differ significantly between groups (Table 2).

**Table 2:** Post operative outcomes "+" denotes Fisher’s exact test. Categorical variables were compared using Pearson's chi-square test when the assumptions for the test were met. Fisher's exact test was used when expected cell frequencies were less than 5. SSI, surgical site infection; n, number; DVT, deep venous thrombosis; PE, pulmonary embolism; MI, myocardial infarction

Type of surgery & outcomes, n(%)	Total (n = 70)	Vessiloop (n = 35)	No Vessiloop (n = 35)	p-value
Emergency cases	68 (97.1)	34 (97.1)	34 (97.1)	1.000^+^
Elective cases	2 (2.9)	1 (2.9)	1 (2.9)	
Type of surgery				
Colorectal surgery	26 (37.1)	12 (34.3)	14 (40.0)	0.621
Small bowel surgery	41 (58.6)	22 (62.8)	19 (54.3)	0.467
Hernia surgery	1 (1.4)	0 (0.0)	1 (2.8)	1.000^+^
Upper gastrointestinal surgery	1 (1.4)	0 (0.0)	1 (2.8)	1.000^+^
Other surgery	1 (1.4)	1 (2.8)	0 (0.0)	1.000^+^
Outcomes				
SSI	6 (8.6)	0 (0.0)	6 (17.1)	0.025^+^
- Superficial	5 (7.1)	0 (0.0)	5 (14.2)	-
- Deep	0 (0.0)	0 (0.0)	0 (0.0)	-
- Organ/space	1 (1.4)	0 (0.0)	1 (2.8)	-
Pneumonia	1 (1.4)	1 (2.8)	0 (0.0)	1.000^+^
Sepsis	0 (0.0)	0 (0.0)	0 (0.0)	-
Bleeding	5 (7.1)	3 (8.6)	2 (5.7)	1.000^+^
DVT	1 (1.4)	0 (0.0)	1 (2.8)	1.000^+^
PE	0 (0.0)	0 (0.0)	0 (0.0)	-
MI	2 (2.8)	0 (0.0)	2 (5.7)	0.493^+^
Return to the theatre	2 (2.9)	0 (0.0)	2 (5.7)	0.493^+^
Palliation	3 (4.2)	1 (2.8)	2 (5.7)	1.000^+^
Death (30-day)	3 (5.7)	1 (5.7)	2 (5.7)	1.000^+^
Readmission (30-day)	4 (5.7)	1 (2.8)	3 (8.6)	0.614^+^
Wound class				
- Clean	10 (14.3)	3 (8.6)	7 (20.0)	0.003^+^
- Clean - contaminated	41 (58.6)	18 (51.4)	23 (65.7)	
- Contaminated	5 (7.1)	1 (2.9)	4 (11.4)	
- Dirty	14 (20.0)	13 (37.1)	1 (2.9)	

No SSI occurred among patients in the Vessiloop group (0.0%; 95% CI 0.0-10.0%) compared with six patients in the conventional closure group (17.1%; 95% CI 6.6-33.6%); Fisher’s exact test, p = 0.025. Baseline differences between groups may have contributed to the observed SSI rates. Most infections were superficial, with only one organ/space infection identified.

An analysis of factors associated with SSI demonstrated a relationship between SSI development and smoking. Other comorbidities, including diabetes, obesity, and cardiovascular disease, were not significantly associated with SSI in this cohort. These findings suggest that smoking appeared to be associated with SSI (p = 0.034); the small number of smokers limits the reliability of this observation. These findings should be considered exploratory and require confirmation in larger studies (Table 3).

**Table 3:** Association of surgical site infection (SSI) with type of surgery and comorbidities "+" denotes Fisher’s exact test. All categorical variables were calculated with Fisher’s exact test. UGI, upper gastrointestinal; DVT, deep venous thrombosis; ASA, American Society of Anesthesiologists; CKD, chronic kidney disease; PVD, peripheral vascular disease

Type of surgery	Total n = 70 (%)	SSI group n = 6 (%)	p-value
Colorectal	26 (37.1)	1 (16.6)	0.401
Small bowel	41 (58.6)	4 (66.6)	1.000
Hernia	1 (1.4)	0 (0.0)	1.000
UGI	1 (1.4)	1 (16.6)	0.086
Other Surgery	1 (1.4)	0 (0.0)	1.000
Comorbidities			
Diabetes	11 (15.7)	2 (33.3)	0.236
Cardiovascular disease	20 (28.5)	2 (33.3)	1.000
Hypertension	30 (42.8)	3 (50.0)	1.000
Dyslipidaemia	15 (21.4)	0 (0.0)	0.329
Chronic kidney disease	5 (7.1)	1 (16.6)	0.370
Liver disease	2 (2.8)	1 (16.6)	0.165
Chronic lung disease	8 (11.4)	0 (0.0)	1.000
Peripheral vascular disease	1 (1.4)	0 (0.0)	1.000
Neurological disease	7 (10)	0 (0.0)	1.000
Cancer	13 (18.5)	0 (0.0)	0.585
Obesity	2 (2.8)	1 (16.6)	0.165
GI disease	22 (31.4)	3 (50.0)	0.370
Previous abdominal surgery	19 (27.1)	2 (33.3)	0.660
DVT history	1 (1.4)	0 (0.0)	1.000
Steroid use	1 (1.4)	1 (16.6)	0.086
Immunosuppression	13 (18.5)	3 (50.0)	0.073
Smoker	4 (5.7)	2 (33.3)	0.034

## Discussion

This study demonstrates an association between vessel loop-assisted skin closure and a reduced incidence of incisional SSI following laparotomy. No SSIs were observed in patients treated with Vessiloop, compared with a 17.1% incidence in those undergoing conventional closure.

Vessel loops have been widely used in other surgical contexts, including abscess drainage and delayed closure following fasciotomy. Previous studies have shown that vessel loop techniques are technically simple, well tolerated, and associated with high patient satisfaction due to reduced discomfort during removal; in addition, this technique did not increase complications or suturing time [10,11]. A case series related to fasciotomy for compartment syndrome secondary to crush injury that used Vessiloop for wound closure found that the average wound closure time was three weeks without any complications, such as SSI [10,12]. Our findings extend these observations to laparotomy closure.

The economic implications of SSI are substantial. With an average cost exceeding AUD $18,000 per case, even modest reductions in SSI incidence may yield significant cost savings [6]. In contrast, the cost of vessel loops is minimal, suggesting a favourable cost-benefit profile [10].

There is a strong relationship between comorbidities and the development of SSI. A large retrospective study found that diabetes, liver disease, renal failure, malnutrition/weight loss, obesity, cardiac arrhythmias, depression, and chronic pulmonary disease were strongly associated with SSI. Similar findings were reported by Aga et al., who also observed that immunosuppressive treatment and ASA > 2 were associated with an increased risk of SSI [1,13]. In our cohort, smoking was strongly associated with SSI, consistent with prior literature. These findings reinforce the importance of targeted SSI prevention strategies in high-risk patient populations.

Microbial contamination is a well-known risk factor for SSI. In open laparotomy, especially in gastroenterological surgery, pathogens are often introduced from the patient’s skin and hollow viscera, from the surgical team and surgical instruments, or from the air [4]. According to Watanabe et al. and Aga et al., the incidence of SSI in patients who undergo colorectal surgery can be as high as 20% [1,14]. In contrast, Isozaki found that gastrointestinal surgery has an incisional SSI rate of 10% or higher, often occurring during colorectal surgery or during abdominal cavity contamination [15].

In our study, gastrointestinal surgery was associated with a higher incidence of SSI, similar to previous studies. The proposed mechanism underlying the observed reduction in SSI relates to passive drainage of subcutaneous fluid, thereby reducing dead space and limiting bacterial proliferation. This principle of subcutaneous fluid drainage is similar to previous studies that used closed suction drainage to remove fluid from the laparotomy wound, demonstrating reduced SSI rates following abdominal surgery [13,16,17].

Strengths and limitations

This study has several strengths. Vessiloop is a simple and inexpensive surgical technique that can be readily implemented in routine clinical practice without specialised equipment or additional operative time. Furthermore, the analysis and inclusion of patients undergoing a broad range of abdominal procedures reflect real-world surgical practice, enhancing the clinical relevance of the results. The study also takes into consideration patient-related risk factors for SSI, especially smoking, providing additional insight into factors influencing postoperative wound complications in our population.

However, several limitations should be acknowledged. Its retrospective design and single-centre setting limit generalisability. The choice of wound closure technique was based on surgeon preference rather than random allocation, introducing potential selection bias. The conventional closure group was older and had a higher ASA classification, which may have influenced the observed outcomes. Several perioperative variables associated with SSI risk were not available in this retrospective cohort and were not included in the analysis. The small number of SSI cases limited the study's statistical power and prevented a multivariable regression analysis to adjust for potential confounders. Larger prospective studies are required to confirm these findings.

## Conclusions

In this retrospective cohort study, subcutaneous Vessiloop application during laparotomy wound closure was associated with a lower incidence of SSI compared with conventional skin closure. However, considering this is an observational study, baseline differences between groups, the small number of SSI events, and the potential for residual confounding, these findings should be considered hypothesis-generating. Larger prospective randomised controlled trials are warranted to confirm these findings and validate the role of this technique in routine surgical practice.
